# Endemic within endemics: the microbiota of the Galapagos marine iguanas

**DOI:** 10.1093/ismeco/ycag040

**Published:** 2026-03-09

**Authors:** Ido Grinshpan, Omer Lavy, Alvah Zorea, Itai Amit, Liron Levin, Ori Furman, Daniel Somekh, Nataly Guevara, Sarah Moraïs, Otto X Cordero, Itzhak Mizrahi

**Affiliations:** Department of Life Sciences, Ben-Gurion University of the Negev, Beer-Sheva 84105, Israel; National Institute for Biotechnology in the Negev (NIBN), Ben-Gurion University of the Negev, Southern District Beer-Sheva 84105, Israel; School of Sustainability and Conservation, Ben-Gurion University of the Negev, Beer-Sheva, Southern District 84105, Israel; Department of Life Sciences, Ben-Gurion University of the Negev, Beer-Sheva 84105, Israel; National Institute for Biotechnology in the Negev (NIBN), Ben-Gurion University of the Negev, Southern District Beer-Sheva 84105, Israel; School of Sustainability and Conservation, Ben-Gurion University of the Negev, Beer-Sheva, Southern District 84105, Israel; Department of Life Sciences, Ben-Gurion University of the Negev, Beer-Sheva 84105, Israel; National Institute for Biotechnology in the Negev (NIBN), Ben-Gurion University of the Negev, Southern District Beer-Sheva 84105, Israel; School of Sustainability and Conservation, Ben-Gurion University of the Negev, Beer-Sheva, Southern District 84105, Israel; Department of Life Sciences, Ben-Gurion University of the Negev, Beer-Sheva 84105, Israel; National Institute for Biotechnology in the Negev (NIBN), Ben-Gurion University of the Negev, Southern District Beer-Sheva 84105, Israel; School of Sustainability and Conservation, Ben-Gurion University of the Negev, Beer-Sheva, Southern District 84105, Israel; Bioinformatics Core Facility, llse Katz Institute for Nanoscale Science and Technology, Ben-Gurion University of the Negev, Beer-Sheva, Southern District 84105 Israel; Department of Life Sciences, Ben-Gurion University of the Negev, Beer-Sheva 84105, Israel; National Institute for Biotechnology in the Negev (NIBN), Ben-Gurion University of the Negev, Southern District Beer-Sheva 84105, Israel; School of Sustainability and Conservation, Ben-Gurion University of the Negev, Beer-Sheva, Southern District 84105, Israel; Department of Life Sciences, Ben-Gurion University of the Negev, Beer-Sheva 84105, Israel; National Institute for Biotechnology in the Negev (NIBN), Ben-Gurion University of the Negev, Southern District Beer-Sheva 84105, Israel; School of Sustainability and Conservation, Ben-Gurion University of the Negev, Beer-Sheva, Southern District 84105, Israel; Galapagos Science Center, Universidad San Francisco de Quito, Puerto Baquerizo Moreno, Isla San Cristóbal 200150, Ecuador; Department of Life Sciences, Ben-Gurion University of the Negev, Beer-Sheva 84105, Israel; National Institute for Biotechnology in the Negev (NIBN), Ben-Gurion University of the Negev, Southern District Beer-Sheva 84105, Israel; School of Sustainability and Conservation, Ben-Gurion University of the Negev, Beer-Sheva, Southern District 84105, Israel; Department of Civil and Environmental Engineering, Massachusetts Institute of Technology (MIT), Cambridge, MA 02139, United States; Department of Life Sciences, Ben-Gurion University of the Negev, Beer-Sheva 84105, Israel; National Institute for Biotechnology in the Negev (NIBN), Ben-Gurion University of the Negev, Southern District Beer-Sheva 84105, Israel; School of Sustainability and Conservation, Ben-Gurion University of the Negev, Beer-Sheva, Southern District 84105, Israel

**Keywords:** microbial ecology, host–microbe interactions, community assembly, neutral theory, purifying selection, endemism, marine iguana, gut microbiota, phylosymbiosis, *Clostridia*

## Abstract

The ecological processes shaping host-associated microbial communities in geographically isolated ecosystems remain poorly understood—particularly the interplay between dispersal, selection, and microbial speciation. Here, we characterize the fecal microbiota of the Galápagos marine iguana (*Amblyrhynchus cristatus*), an iconic endemic vertebrate that depends on its microbiota to digest an algae-based diet. We analyzed fecal samples from 111 individuals across three remote colonies and found that fecal microbial composition is dominated by *Clostridia*, closely following a neutral dispersal model. Yet, ecological and phylogenetic analyses revealed novel, host-restricted *Clostridia* clades—spanning species to family level—that appear to have diversified within marine iguanas. These lineages are consistently retained across host populations through strong purifying selection, resulting in striking microbiota homogenization. Our findings demonstrate that endemic hosts can support microbially distinct lineages shaped by stochastic dispersal and parallel selection, advancing our understanding of microbial community assembly in obligate host–microbiota systems.

## Introduction

The endemic species of the Galapagos [1] established and developed in this unique habitat over millions of years and drew the interest of a plethora of researchers for hundreds of years, including Charles Darwin [2]. Despite extensive research on the ecology and evolution of endemic vertebrates and invertebrates in the Galapagos archipelago, the analysis and characterization of the microbial communities associated with these species have only been recently initiated in the past decade [3–5]. To date, there are still substantial gaps in our understanding of host–microbiota relationships in these unique animals. The existence of geographically isolated microbial ecosystems offers a natural framework for investigating broad ecological processes such as microbial selection, dispersal, and diversification within these relatively closed systems [6,7]. This framework could provide a basis for addressing fundamental questions that remain unanswered, for instance, do the microbial communities residing within endemic hosts also contain endemic microbial species shaped by geographic isolation? And how do ecological and evolutionary forces influence the structure and function of these communities? Additionally, given the crucial ecological roles of microbial communities in host health and ecosystem stability, how can we preserve microbiota diversity alongside host species [8–10]?

To explore these questions, we focused on the microbiota of the marine iguanas (*Amblyrhynchus cristatus*), the world’s only sea-going lizards and one of the most iconic endemic species of the Galapagos Islands. Notably, these species have an obligate symbiosis with their fecal microbiota. Indeed, while these ectothermic herbivores feed exclusively on green and red algae found in intertidal and subtidal zones, relying on polysaccharides such as agar and cellulose as their primary energy sources [11], they lack the enzymes required to degrade these complex polymers, therefore relying entirely on their fecal microbiota to break them down and convert them into usable chemical energy, forming an obligate symbiosis [5,12]. Hence, the marine iguanas present a unique opportunity to investigate an obligate host–microbiota system within an endemic species confined to a specific geographic region. Furthermore, marine iguanas inhabit colonies distributed across the archipelago. While migration occurs between some of these populations, especially in the western islands such as Fernandina, strong genetic structure is maintained per island, effectively creating an “islands within islands” scenario [13,14]. This system—composed of distinct populations connected by occasional dispersal—provides an ideal framework for examining the balance between stochastic processes and selection in shaping microbial communities [15–18]. Dispersal, selection, speciation, and drift are the principal processes structuring microbial communities. In intestinal ecosystems, drift is generally considered less influential due to large population sizes and strong host filtering, though it may still impact low-abundance taxa [16,19–21]. Building on this framework, we investigated the compositional dynamics of marine iguana fecal microbiotas to address key gaps in our understanding of how microbial communities assemble in geographically isolated, host-associated systems. We collected fecal samples from 111 individuals across two islands and three distinct colonies in the Galápagos archipelago. Using 16S ribosomal RNA (16S rRNA) gene sequencing, ecological modeling, and phylogenetic analysis, we found that these communities are dominated by members of the *Clostridia* class and contain unique, host-restricted taxa—extending up to the family level—that appear to be diversifying within the host lineage.

To interpret these patterns, we applied the neutral model of metacommunity assembly, which assumes functional equivalence and stochastic dispersal [19, 20, 22]. Widely used in microbial ecology studies, this model served here both as a testable hypothesis and as a null expectation against which the effects of selection and diversification could be evaluated. While the overall community structure aligned with neutral predictions, we also found strong evidence of purifying selection: specific microbial clades that exhibit less diversification than expected were observed more frequently than expected. These findings suggest that although dispersal drives widespread colonization, shared selective pressures across hosts act to homogenize microbiota composition and maintain functionally important lineages, indicating a dynamic balance between neutral processes and host-driven selection in shaping microbial communities.

## Materials and methods

### Sample collection

Fecal samples were collected in the Galapagos Islands from three areas: Punta Espinosa and Cabo Douglas on Fernandina Island, and Rabida Island. During sample collection, the coordinates of each location were recorded. Samples were collected from the rocks immediately after defecation into 2 ml cryotubes and were quickly frozen in liquid nitrogen. On Rabida Island, eight lizards were captured, and samples were collected using anal swabs. Samples were kept at −80°C until processing.

### DNA extraction

200 mg of feces were suspended in 500 μl of Tris-EDTA (TE) buffer (10 mM Tris–HCl, 1 mM EDTA). Suspended samples were mechanically lysed with phenol using bead beating, followed by phenol/chloroform extraction. The final supernatant was incubated overnight at −20°C with 0.1 volume of 3.0 M sodium acetate and 0.6 volume of isopropanol. The following day, samples were pelleted at 16 000 × g for 30 min at 4°C and resuspended in 1 ml of 70% ethanol. Samples were then pelleted again, the liquid was aspirated, and the samples were left to dry. Finally, the samples were resuspended in 50 μl of TE buffer and kept frozen at −20°C.

### 16 s rRNA amplicon sequencing

The amplification of the V4 region of the 16S rRNA gene from fecal samples followed the method outlined by the Earth microbiome project [23–25]. In short, Primers 515F and 806R were used, with each reverse primer containing a distinct 12-bp index. The amplification protocol consisted of an initial denaturation step at 94°C for 15 min, followed by 35 cycles of denaturation at 94°C for 45 s, annealing at 50°C for 60 s, and extension at 72°C for 90 s, with a final extension step at 72°C for 10 min. PCR products were purified using the DNA Clean & Concentrator kit™-5, from Zymo Research and quantified for Illumina adapter-containing fragments using quantitative polymerase chain reaction (qPCR). Sequencing was conducted on the Illumina Miseq platform. Subsequently, samples were diluted to a concentration of 4 nM and prepared for sequencing following the manufacturer’s instructions. The normalized samples were then pooled and subjected to paired-end sequencing.

### 16 s rRNA analysis using Quantitative Insights Into Microbial Ecology (QIIME), subsampling reads, filtering erroneous reads

Sequences were processed using the QIIME 2 pipeline [24], sequences were imported using the Earth Microbiome Project parameters for paired end sequences and demultiplexed to remove barcodes and adaptors. Demultiplexed paired end sequences were then subjected to DADA2 quality filtering [26] following open reference taxonomy annotation using the SILVA 138 database [27]. To avoid amplicon sequence variant (ASV) inflation and biases due to uneven sampling depth, common issues in amplicon-based studies, the samples were subsampled to a depth of 5300 reads using the rarefy_even_depth R function in the Phyloseq package [28] and only ASVs that had more than two reads in over 5% of the samples were retained [29]. This approach minimized sequencing artifacts and ensured a more accurate representation of the microbial communities.

### Alpha and beta-diversity analysis

All downstream ecological statistics were carried out in R 4.3.3. Alpha-diversity metrics (ASV richness, Shannon index, Faith’s phylogenetic diversity [30]) were computed with Phyloseq v1.46. Beta diversity was quantified using weighted and unweighted UniFrac and Bray–Curtis dissimilarities. Community clustering was visualized by principal-coordinate analysis (PCoA). Significance of among-site differences was tested with (i) ANOSIM (999 permutations, vegan v2.6–4) [31]. *P*-values were FDR-corrected where multiple tests were conducted.

### Neutral community model and ecological selection

The distribution of ASVs per iguana population was analyzed using a modified neutral community model (NCM) [32] that considers the large population sizes typical of prokaryotic communities and integrates competitive advantages [33]. Our investigation focused on assessing the extent to which the abundance patterns of ASVs align with this modified near-neutral model, and which ASVs deviate from the neutral model. The parameter mN represents the metacommunity size multiplied by the immigration rate. Deviations from neutrality were tested with two-tailed exact binomial tests treating each taxon’s presence/absence profile as N Bernoulli trials with success probability F_fit,i; *P* < .05 indicated significant deviation. Taxa with F_obs,i > F_fit,i were labelled “Ecologically selected,” those with F_obs,i < F_fit,i “Not ecologically selected,” and all others “Neutral.” This provided a reproducible partition of taxa into neutrally assembled dispersal-limited or selectively enriched groups.

### Random-forest classification of sampling location

To identify fine-scale compositional signatures, we trained a random-forest (RF) model (randomForest v4.7–1.1 [34]) using centered-log-ratio (CLR) transformed ASV abundances. The data set was stratified by site and split 80/20% into training and test sets, maintaining class balance. Performance was summarized as overall accuracy and class-specific sensitivity/specificity. Variable importance was computed by mean decrease in accuracy.

### *Clostridia* tree construction, clustering analysis, comparison to terrestrial iguana

We composed a phylogenetic tree of *Clostridia* from 3365 16S rRNA sequences existing in both the SILVA and Greengenes databases. These sequences were aligned using MAFFT [35] and ordered into a phylogenetic tree with fasttree (v2.1.11) [36] using the -nt flag and default settings. To observe whether marine iguanas Clostridium clusters more than other sequences on the tree, we have iteratively (1000 iterations) sub-sampled 50 sequences and calculated mean phylogenetic distances within those groups, then compared the 1000 mean phylogenetic distances generated.

### Comparative analysis with Fiji terrestrial iguanas

To contextualize our findings within a broader phylogenetic framework, we downloaded raw 16S rRNA gene amplicon sequencing data from a previously published cohort of Fiji terrestrial iguanas [37]. We ensured that the Fiji cohort was sequenced using the same primer set targeting the V4 hypervariable region (515F/806R) and the same sequencing platform (Illumina MiSeq) as our current study. To eliminate bioinformatic artifacts arising from differing software versions or parameter choices, raw FASTQ files from the Fiji cohort were re-processed *de novo* using the exact same analytical pipeline as our Galapagos dataset (described above). This included identical quality filtering, error correction/denoising, and taxonomic assignment steps, ensuring that all ASVs were generated and filtered under uniform conditions.

### Statistical robustness and additional compositional analysis

All statistical analyses were performed in R v4.3.3 [38]. To evaluate sampling saturation, species accumulation curves were generated using a custom bootstrapping function with 100 random permutations per location. To address compositional bias, raw read counts were centered log-ratio (CLR)–transformed using decostand (method “clr”) after adding a pseudo-count. Beta diversity was re-assessed using Aitchison distances (Euclidean distance on CLR-transformed data) and visualized via Principal Coordinate Analysis (PCoA). To ensure that observed community differences were driven by compositional turnover rather than heterogeneous variance, multivariate homogeneity of group dispersions was assessed using betadisper in vegan v2.6–4. Differences in dispersion between locations were tested using a permutation-based test (permutest, 999 permutations). Statistical significance of geographic clustering was further confirmed using ANOSIM (999 permutations) on the Aitchison distance matrix. Finally, to verify that Random Forest classification was not driven by class imbalance, we implemented an iterative down-sampling approach where larger groups were randomly subsampled to match the minority class (*n* = 8) across 1000 retraining iterations, evaluating performance based on mean accuracy (Fig. S4).

### Diversification estimation of *Clostridia* in marine Galapagos iguanas

We used the phylogenetic tree of *Clostridia* to calculate pairwise phylogenetic distances among all sequences. For each *Clostridia* ASV derived from marine iguanas, we calculated phylogenetic distance to the nearest non-iguana sequence. We then counted the number of neighboring sequences within the iguana-derived clade that were phylogenetically closer to the focal sequence than its closest non-iguana relative. To establish a null expectation, we randomly sampled sequences and phylogenetic distances 1000 times, generating a null model describing the relationship between phylogenetic distance and the expected number of neighbors by fitting a nonlinear equation. Using this model, we calculated the expected number of neighbors for each clade based on its branch age. The diversification index was defined as the difference between the observed and expected number of neighbors, representing the degree of excess or reduced diversification relative to the null expectation.

## Results

### Sampling regime across the Galapagos archipelago

**Figure 1:**
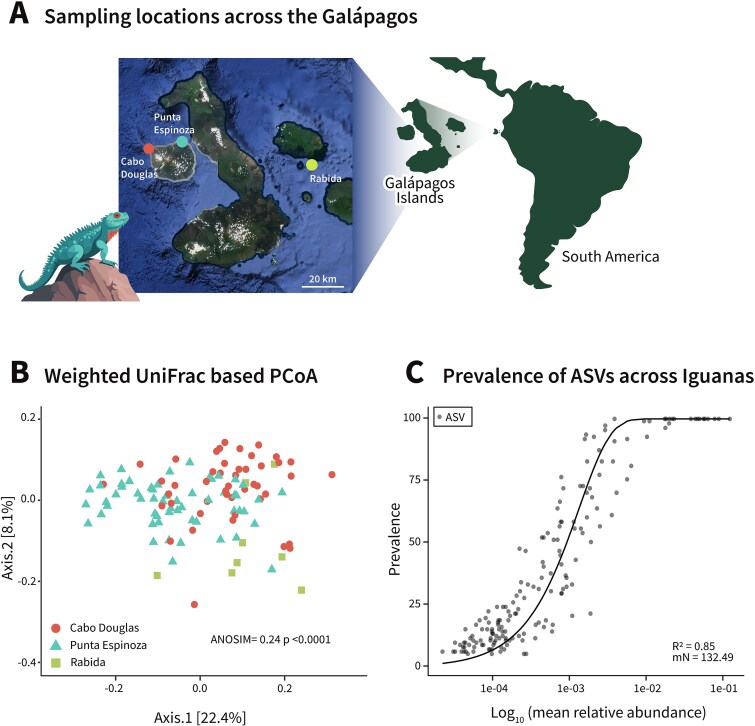
The Galapagos marine iguanas’ microbiota is governed by neutral dispersal of microbes. (A) Location of the three sampling sites and their respective sample sizes: Punta Espinoza (PE, *n* = 57), Cabo Douglas (CD, *n* = 47), and Rabida (RA, *n* = 8). Dot size reflects the number of individuals sampled per colony. (B) Visualization of microbial community beta diversity via PCoA, utilizing weighted UNIFRAC distances, sample locations are shape-coded as depicted in the figure. ANOSIM analysis indicated a weak geographical clustering (*R* = 0.245, *P* < .001). (C) A graphical depiction of the neutral dispersal through all the sampling locations model shows a strong positive relationship between microbial abundance and their prevalence across individual iguanas (*R*^2^ = 0.85, mN = 132.49). Each dot represents an amplicon sequence variant (ASV) identified in the fecal microbiota of Galápagos marine iguanas.

To investigate the marine iguana's microbiota, we sampled fecal pellets from 111 individual iguanas across three locations on two Galapagos Islands: Fernandina and Rábida (Fig. 1A). On Fernandina Island, sampling was conducted at two distinct sites—Punta Espinoza on the eastern coast and Cabo Douglas on the western coast. These sites are ~20 km apart and are inhabited by separate iguana populations. Given the physical isolation of the Galapagos Islands and the relative geographical separation between iguana colonies, we first examined the occurrence and dispersal patterns of microbial species within and between populations across the different sampling sites.

### Neutral dispersal governs the microbial communities’ composition of the Galapagos marine iguanas

We hypothesized that the geographical barriers between iguana colonies, combined with the known limitations on individual dispersal, would restrict microbial exchange and lead to distinct clustering of fecal microbial communities according to sampling sites. However, a beta diversity analysis of the microbiotas across these geographically distinct locations revealed only subtle geographic clustering (Fig. 1B), despite the considerable distances separating the islands and their iguana populations (Fig. 1A). Specifically, an analysis of similarity (ANOSIM) based on weighted UniFrac distances yielded a low *R* value of 0.245. Although statistically significant (*P* < .001), this value falls below the threshold typically considered indicative of meaningful biological clustering [39]. Additionally, permutational multivariate analysis of variance (PERMANOVA) confirmed significant compositional differences between sites (*R*^2^ = 0.245, *P* < .001), while beta dispersion analysis showed no significant difference in within-group variance (*P* = .745), confirming that clustering is driven by compositional turnover rather than dispersion artifacts (Fig. S1). Given the unexpected limited differentiation between microbial communities across colonies, we hypothesized that ecological and evolutionary forces may be contributing to the homogenization of the marine iguana fecal microbiota. To explore this hypothesis, we first tested the simplest explanatory model of the neutral metacommunity paradigm. According to this model, community assembly is governed by demographic stochasticity alone, predicting a direct relationship between the abundance of microbes in the overall species pool (gamma diversity) and the number of hosts in which they are found, independent of geography or dispersal limitations. Our analysis revealed a strong fit between the observed microbial distribution and the neutral model’s predictions (Fig. 1C), with an *R*^2^ value of 0.85. Notably, this neutrality was also observed within each iguana population (Fig. S2). This suggests that the microbial species disperse in a largely neutral manner across individual marine iguanas, resulting in high community similarity across habitats. These findings indicate that, despite considerable geographic separation, including aerial distances exceeding 20 km between Punta Espinoza (PE) and Cabo Douglas (CD), and the isolation of Fernandina from Rábida, microbial prevalence is primarily driven by overall species abundance rather than spatial constraints. Nevertheless, while our results highlight neutral processes as a dominant force shaping these communities, they do not exclude the influence of additional ecological factors. In particular, strong, consistent, and homogeneous selective pressures across habitats—such as the marine iguanas’ highly uniform algae-based diet.

### *Clostridia* amplicon sequence variants homogenize the marine iguana microbiota yet reveal site-specific signatures

**Figure 2:**
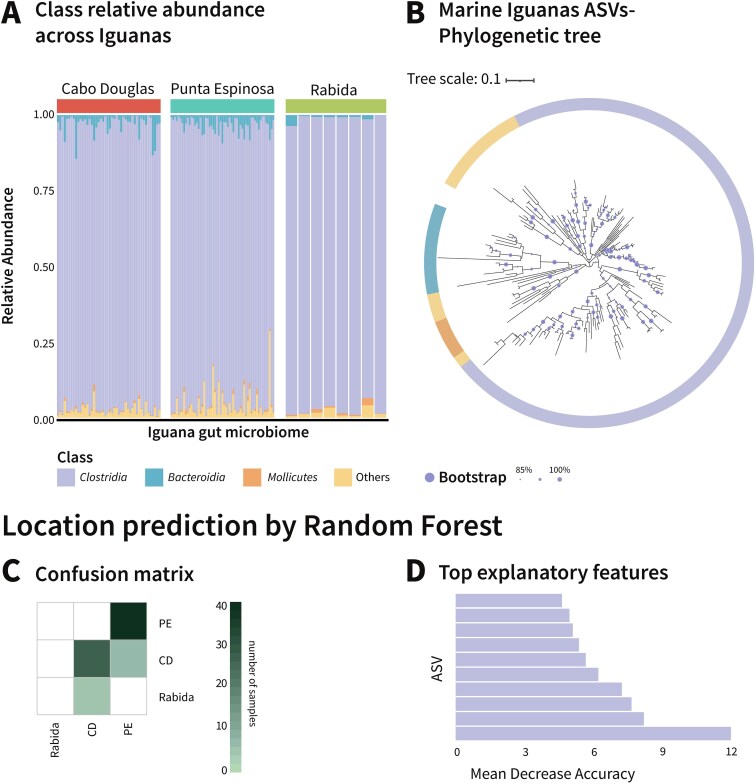
*Clostridia* dominate the Galapagos marine iguana’s microbiota. (A) Relative abundance taxonomic bar plots of all iguanas’ microbiotas, separated on the y axis by the phylogenetic class and separated on the x axis by the sampling location. *Clostridia* class represents up to 80%–99% of the microbial abundance in most individuals. (B) Phylogenetic tree of all 176 ASVs discovered in this study, separated by the class,122/176 of ASVs being classified as *Clostridia*. (C) Random Forest confusion matrix of the classifications of the iguana’s location based on the microbiota composition, with 5–10 erroneous samples’ classification at each model run iteration. The gradient represents the number of samples the number of samples predicted, with rows being the true location, and the columns being the predicted location. Overall, the model had a mean location prediction accuracy of 87.5%. (D) The top 10 amplicon sequence variants (ASVs) identified by a random forest classifier trained to predict iguana sampling location based on fecal microbiota composition. All selected ASVs belong to the *Clostridia* class.

To further investigate the possibility of homogeneous selection acting on the marine iguana fecal microbiota, we first analyzed taxonomic composition across the sampling sites. This analysis revealed that the class *Clostridia* overwhelmingly dominates the marine iguana fecal microbiota, accounting for 80%–99% of the microbial community across all individuals and locations (Fig. 2A, Fig. S3A). This pattern is consistent with previous reports of *Clostridia* dominance in iguana fecal microbiotas [40]. To assess finer-scale taxonomic variation within these *Clostridia*-dominated communities, we applied a random-forest machine learning algorithm. Remarkably, the model was able to predict the sampling location of a given microbial community with 87.5% accuracy (Fig. 2C). Notably, the top 10 features contributing to the model’s classification were all *Clostridia* amplicon sequence variants (Fig. 2D), suggesting that certain *Clostridia* taxa may be subject to localized selective pressures. These differences were primarily driven by variations in the relative abundance of shared taxa across sites, rather than the presence or absence of site-specific species, except for a single unique ASV. This observation aligns with our clustering analyses, which revealed no clear site-specific taxa restricted to individual locations.

### *Clostridia* from Galapagos marine iguanas form unique phylogenetic clusters

**Figure 3:**
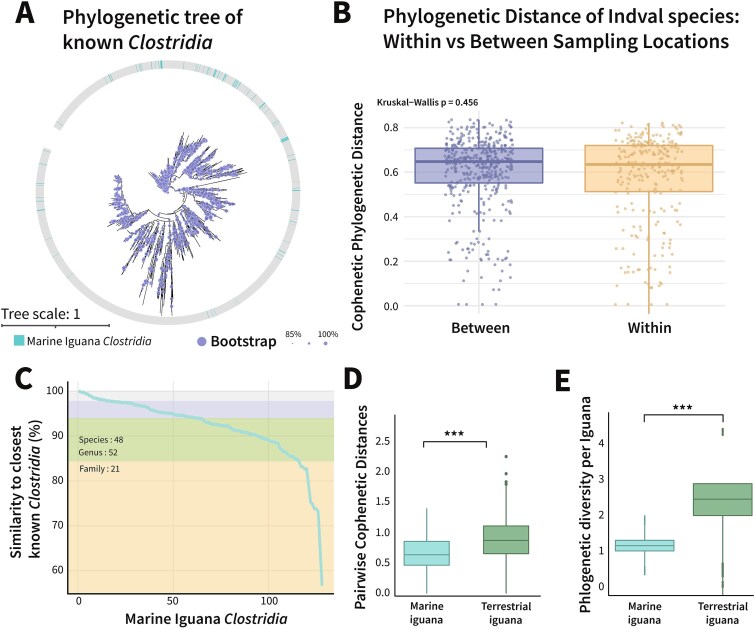
*Clostridia* originating from Galapagos marine iguanas form novel endemic clusters. (A) Phylogenetic tree composed of *Clostridia* 16 s rRNA genes retrieved from GreenGenes and SILVA with the *Clostridia* sequences obtained from marine iguanas and terrestrial iguanas from Fiji. Branches originating from marine iguanas are highlighted. (B) Pairwise phylogenetic cophenetic distances sequences of IndVal ASV, within and between sampling locations, revealed that the IndVal ASVs are not phylogenetically closer to one another within the same location (Kruskal−Wallis *P* = .456). (C) Similarity of each *Clostridia* ASV to the closest known *clostridia* (*y* axis); each dot is an individual ASV. In total, none of the ASVs found in the marine iguanas are 100% similar to any known ASV; 48 ASV are considered different species (<98.7% similarity), 52 a different genus (<94.5% similarity), and 21 a different family (<86.5% similarity). (D) Phylogenetic cophenetic distances of *clostridia* within marine iguana and terrestrial iguanas, *clostridia* from marine iguanas are closer phylogenetically to one another than those from terrestrial iguanas (Kruskal–Wallis *P* < .001*)*. (E) Within individual iguana comparison of phylogenetic diversity between marine iguanas and terrestrial iguanas. The box plots represent the distribution of Faith’s phylogenetic diversity across the individual iguanas, with a significantly smaller phylogenetic diversity found in the marine iguanas (Kruskal–Wallis *P* < .001*)*.

We next asked whether the identified fecal microbiotas of marine iguanas harbor endemic *Clostridia* species that are unique to their endemic host. Specifically, we aimed to determine whether *Clostridia* ASVs from marine iguanas differ from previously known *Clostridia* and whether they form distinct, endemic clades on the global *Clostridia* phylogenetic tree. To address this, we aligned the 16S rRNA gene sequences of the marine iguana *Clostridia* with 3236 known *Clostridia* species from the GreenGenes [41] and SILVA databases, the two largest repositories of 16S rRNA gene sequences, to assess the novelty and phylogenetic placement of these taxa (Fig. 3A).

Our analysis revealed that the fecal microbiotas of marine iguanas indeed harbor novel, endemic *Clostridia* taxa spanning taxonomic levels from species to family. For each marine iguana *Clostridia* ASV, we quantified its phylogenetic distance from the closest known reference sequence. Remarkably, none of the 128 *Clostridia* ASVs had 100% identity to any known *Clostridia* (while adhering to the widely accepted taxonomic thresholds proposed by Yarza *et al*. [42]). Suggesting that among them, 48 ASVs fell within the novel species range (98.7%–94.5% similarity), 52 were classified as potentially novel genera (94.5%–86.5% similarity), and 21 were divergent enough to be considered novel families (<86.5% similarity) (Fig. 3C). To further evaluate the uniqueness of these taxa, we compared the *Clostridia* ASVs from marine iguanas to those from terrestrial Fijian iguanas [37]. Pairwise phylogenetic distances among *Clostridia* ASVs within marine iguanas were significantly smaller than those observed in Fijian land iguanas (Fig. 3D, Kruskal–Wallis *P* < .001*)*, indicating tighter phylogenetic clustering. In addition, the per-individual phylogenetic diversity was significantly lower in marine iguanas compared to their terrestrial counterparts (Fig. 3E, Kruskal–Wallis *P* < .001*)*, suggesting stronger selective pressures shaping these communities. Notably, many of these endemic taxa formed unique, host-specific clusters, suggesting clonal diversification within the marine iguana. Together, these findings indicate that selective forces, likely acting at the ecosystem level, are shaping the phylogenetic structure of *Clostridia* in the Galapagos. We hypothesize that this may be driven by the marine iguanas’ highly uniform, algae-based diet, which imposes limited metabolic niche space. This, in turn, may increase competition, constrain diversification, and promote clonal expansion of specific taxa within the marine iguanas.

### Purifying selection leads to retention of specific amplicon sequence variants in the marine iguana bacterial metacommunity

**Figure 4:**
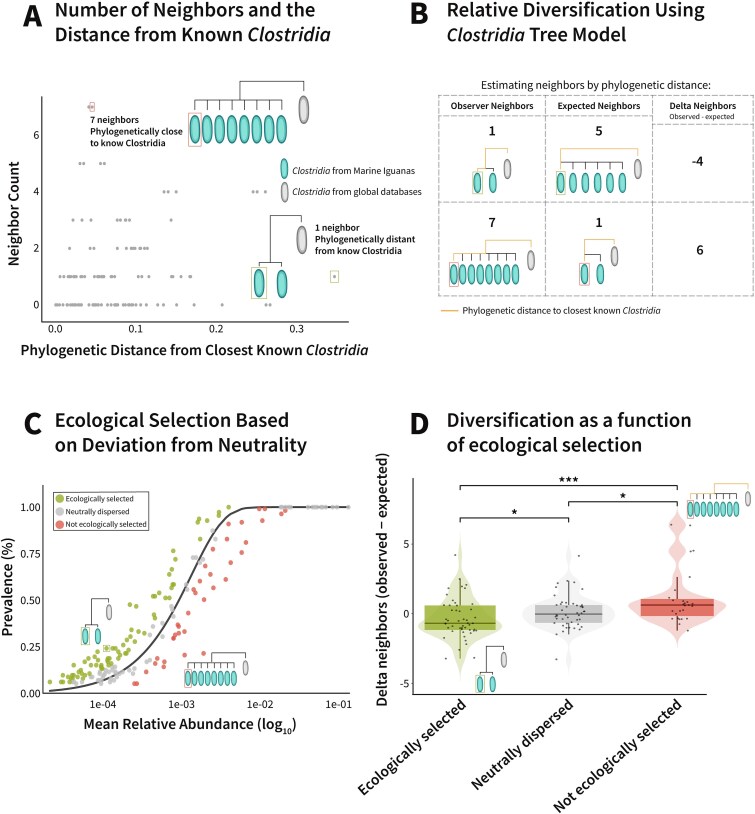
*Clostridia* in marine iguanas undergo parallel ecological selection and phylogenetic purification. (A) Relationship between each ASV’s phylogenetic distance to its closest known relative in the global *Clostridia* reference tree and the number of neighboring *Clostridia* ASVs found in Galápagos samples. This panel illustrates how phylogenetic isolation correlates with local diversification. (B) Calculation of the ‘ASV diversification index’. Each ASV’s phylogenetic distance to its closest global relative was used to estimate the expected number of neighbors, based on the global *Clostridia* phylogeny. The observed minus expected number of neighbors defines the diversification index. Highlighted branches demonstrate the path to the nearest global reference. (C) Neutral dispersal model (as introduced in Fig. 1C), with ASVs classified by their deviation from model expectations. ASVs found at significantly higher frequencies than expected are classified as ‘ecologically selected’; ASVs matching neutral expectations as ‘neutrally dispersed’; and those at significantly lower frequencies as ‘not ecologically selected’. (D) Comparison of diversification indexes among ASVs under different selection regimes. Ecologically selected ASVs show significantly lower diversification indexes compared to nonselected ASVs, including a mean negative value, consistent with purifying evolutionary patterns (Wilcoxon test *P* < .001).

The distinct endemic *Clostridia* ASVs with cohesive phylogenetic patterns in marine iguanas offered a rare opportunity to explore how local environmental pressures and host specificity drive microbial diversification. We therefore investigated the ecological and evolutionary forces shaping the trajectories of these taxa. We hypothesized that the more endemic a bacterial ASV is, the more genetically distinct it would be from global *Clostridia* references, and that greater phylogenetic distinctiveness would reflect increased in-region diversification within the Galapagos. This would be evidenced by a higher number of closely related neighbors among the Galapagos iguana *Clostridia* compared to their nearest non-iguana relatives on the phylogenetic tree. To evaluate this, we calculated, for each marine iguana *Clostridia* ASV, the number of marine iguana *Clostridia* neighbors separating it from its closest non-iguana relative on the phylogenetic tree (Fig. 4A). To determine the relative diversification of the *Clostridia* in the Galapagos to the known *Clostridia*, we constructed a null model based on the global *Clostridia* phylogeny. This model predicted the expected relationship between phylogenetic distance and the number of intervening branches separating any two *Clostridia* ASVs. As expected, the model confirmed that the number of neighboring branches increases with phylogenetic distance. Nevertheless, the marine iguana *Clostridia* did not consistently conform to this pattern. Many *Clostridia* ASVs exhibited fewer neighbors than predicted by the null model constructed from the global *Clostridia* phylogenetic tree. As theory predicts that selection tends to constrain diversity, we hypothesized that the reduced diversity observed within certain marine iguana *Clostridia* clades reflects ecological selection. For each ASV, we calculated the expected prevalence based on its mean abundance and then quantified the deviation from this predicted value [43]. Species found more frequently than expected suggest ecological selection favoring that ASV, while those occurring less frequently than predicted indicate a lack of ecological selection for the marine iguana fecal environment (Fig. 4C). After identifying the selection patterns for each marine iguana *Clostridia* ASV, we compared each ASV’s deviation from the phylogenetic tree model with its corresponding selection pattern. We quantified their deviation from the null phylogenetic model by computing an ASV Diversification Index (ADI). The ADI represents the difference between the observed number of neighboring *Clostridia* ASVs and the number predicted by the null model. For example, if an ASV had two observed neighbors but the model predicted one, its ADI would be +1, indicating more diversification than expected (Fig. 4B). Conversely, if an ASV had three observed neighbors but the model predicted five, the ADI would be −2, suggesting reduced diversification, potentially due to purifying selection. Using this index, we assessed the extent of diversification for each *Clostridia* ASV and tested it against its corresponding selection pattern. Indeed, given the fact that mutations occur randomly, an observation of a sparse phylogenetic neighborhood implies that many intermediate variants have been removed from the population, providing potential evidence of purifying selection and enabling evolutionary inference. In agreement with the initial hypothesis, our results revealed that *Clostridia* species that are ecologically selected in the marine iguana fecal ecosystem had significantly lower Diversification Indices compared to nonselected ones (Fig. 4D) (Wilcoxon test, *P* < .0001). These findings suggest that purifying selection shapes the marine iguana microbiota by favoring specific *Clostridia* ASVs while eliminating others.

## Discussion

Our study provides new insights into the ecological and evolutionary processes shaping microbial communities in geographically isolated ecosystems, using the fecal microbiota of Galapagos marine iguanas (*A. cristatus*) as a model system. We investigated key questions regarding the ecology and evolution of these microbial species, focusing on their dispersal, diversification, and selection. Our results strongly support neutral dispersal as the primary force structuring fecal microbial communities across distinct iguana populations. This observation aligns closely with the metacommunity neutral paradigm, which emphasizes neutral processes such as dispersal and stochastic colonization as central forces shaping microbial community structure across geographically isolated habitats [19,44–47]. This may be partly driven by migration events, particularly in the western archipelago, which act as a potential vector for microbial dispersal [13,18]. A notable ecological finding is the consistent dominance of *Clostridia*, comprising 80%–90% of the microbial community across all sampled locations, showing a strong fidelity between the iguana host and its fecal microbiota. This finding suggests an additional homogenizing force, ecological selection favoring this taxonomic group. While *Clostridia* dominated, we also recovered *Bacteroidia* and *Mollicutes. Bacteroidia* contain known degraders of complex polysaccharides in herbivorous reptiles [5], whereas *Mollicutes* contain common reptile commensals with simplified metabolic capacities [48]. The iguanas’ specialized and uniform algae-based diet requires microbial assistance for fiber degradation, thereby reinforcing a tightly integrated host–microbe association that selects for this taxonomic group. Moreover, the consistency of this diet across islands imposes similar selective pressures in each habitat, promoting parallel community assembly and the repeated selection of the same microbial taxa across individuals, ultimately contributing to the homogenization of fecal microbiota composition. Such diet-driven selective pressures and functional stability of microbiotas have also been observed in human fecal microbiota, demonstrating the broad ecological significance of dietary constraints on microbial communities [49–53].

Additionally, we found clear evidence of selective pressures acting at the level of individual hosts, rather than being driven by geographic location. While overall microbial dispersal among hosts followed a neutral pattern, microbiota components under purifying selection were not clustered or confined to specific localities. These positively selected *Clostridia* ASVs, scattered across sites, exhibited lower diversification indices—consistent with purifying selection, maintaining microbial taxa specifically adapted to the iguanas’ algal diet and digestive physiology. While we acknowledge the limitations of short-read amplicons (V4) for deep evolutionary inference, extensive meta-analyses demonstrate that this region retains sufficient signal to reconstruct host–microbe codiversification and phylosymbiosis patterns [54–56]. Consequently, the strong segregation of ASVs by host population in our data—despite potential neutral dispersal—supports the interpretation that these sequences represent ecologically distinct, host-adapted lineages rather than unstructured noise. These findings indicate that selection pressures are primarily host-associated, likely shaped by the consistent dietary requirements of marine iguanas across the archipelago. Supporting these findings, our phylogenetic analysis revealed distinct clusters of *Clostridia* ASVs exclusive to marine iguanas, which differ significantly from both global *Clostridia* databases and the microbiotas of terrestrial iguanas. Together, these results highlight strong biogeographical and evolutionary structuring driven by host-specific ecological and dietary conditions at the regional community scale.

## Conclusions

Our findings demonstrate the intricate balance between neutral dispersal and host-specific, diet-driven selective pressures in shaping microbial communities of geographically isolated endemic hosts. The identification of endemic microbial lineages underscores the critical role of host-associated ecological factors in microbial evolution and biogeography, while also pointing to a potentially rich reservoir of unexplored metabolic diversity. Importantly, this knowledge emphasizes the need to conserve not only the hosts themselves but also their unique microbial communities, which may be integral to effective conservation strategies.

## Supplementary Material

ycag040_Supplemtary_figures_with_legends_21_1_2026

## Data Availability

Raw sequence reads, sample metadata, and environmental variables have been deposited to the ENA project under PRJEB93862. Custom scripts and datasets required to reproduce the core analysis of this work are available at https://github.com/labmizrahi/Marine_iguanas
