# Supplementary material for: Endemic within endemics: the microbiota of the Galapagos marine iguanas
Source: ISME Commun. 2026 Mar 9;6(1):ycag040. doi: 10.1093/ismeco/ycag040 (PMC13037472; doi:10.1093/ismeco/ycag040)
Supplement: ycag040_Supplemtary_figures_with_legends_21_1_2026 [file ycag040_supplemtary_figures_with_legends_21_1_2026.docx]

**Supplementary figures:**


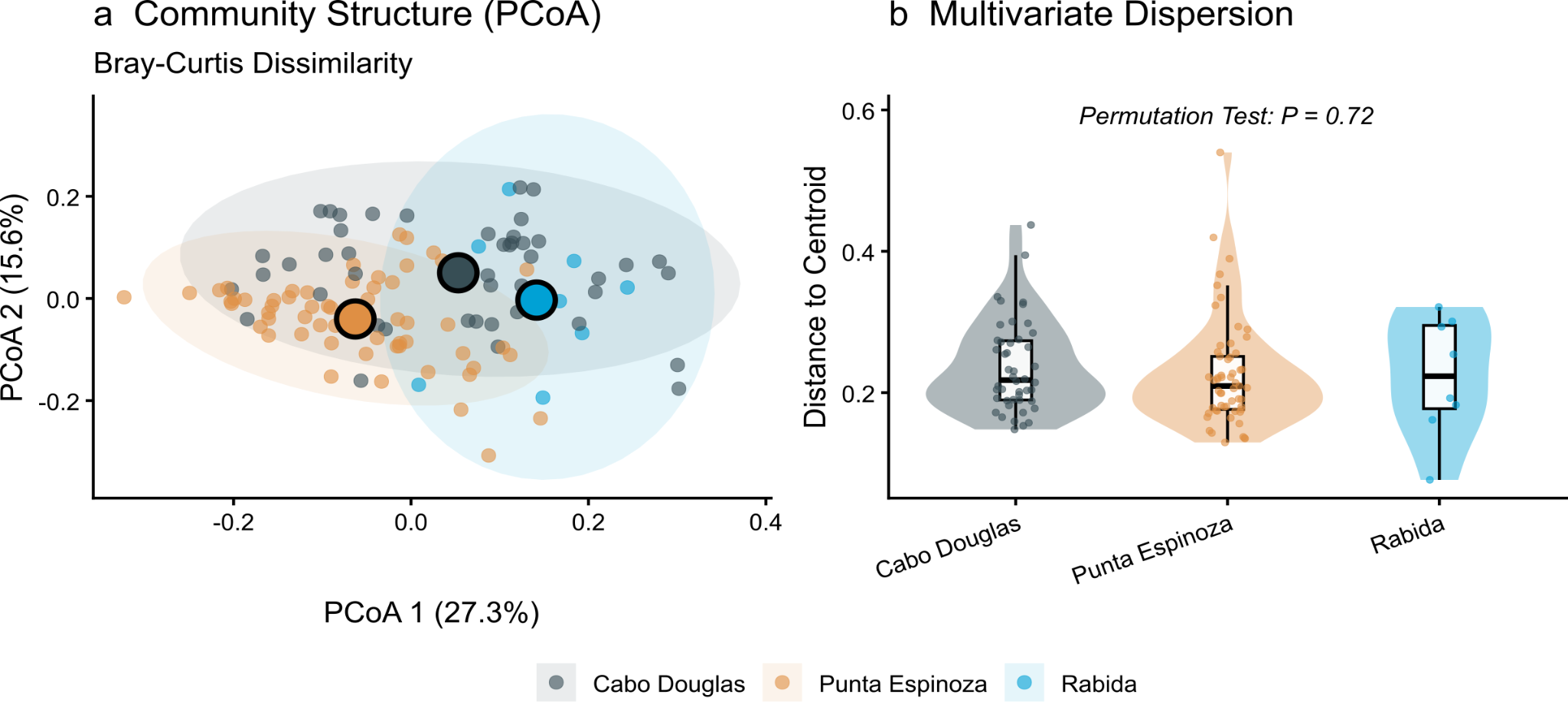

**Figure S1 | Assessment of beta diversity and multivariate dispersion.**

**a.** Principal Coordinate Analysis (PCoA) of Bray-Curtis dissimilarities. Points represent individual samples (n=111); large circles indicate group centroids. Ellipses denote 95% confidence intervals.

**b.** Multivariate homogeneity of group dispersions. Violin plots display the distance of each sample to its group centroid. No significant difference in within-group variance was observed (Permutation test, 999 permutations, P = 0.71), confirming that spatial clustering reflects compositional differences rather than dispersion artifacts.


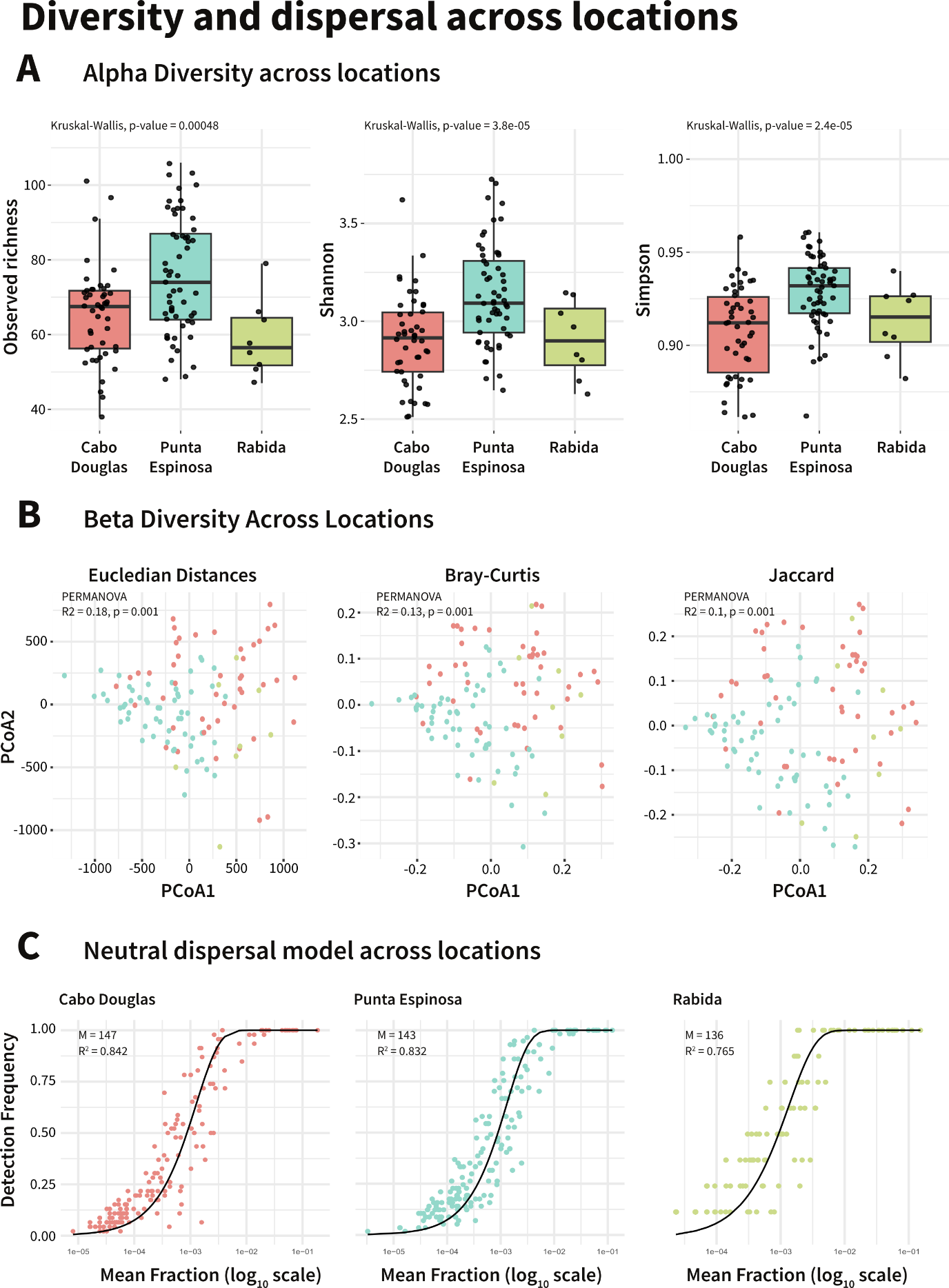


**Figure S2. Marine iguana microbiota alpha diversity and community composition vary across locations, but overall dispersal patterns remain neutral.**

**A.** Alpha diversity (Richness, Shannon index, Jaccard index) of the microbial communities across sample locations. Boxplots show significant variation in within-sample richness between locations (Kruskal–Wallis test *p* < 0.01), with some locations exhibiting higher microbial diversity than others, suggesting local environmental or host-specific filtering, indicating higher alpha diversity at Punta Espinoza compared to both Rabida and Cabo Douglas communities, with no significant differences between both.

**B.** Principal Coordinate Analysis (PCoA) based on Euclidean distances, Bray-Curtis, and Jaccard dissimilarity between samples, colored by sampling location. While some weak clustering by location is visible (R^2=0.18,0.13,0.1 accordingly), there is substantial overlap in microbial community composition, indicating limited geographic structuring.

**C.** Within-location neutral model fitting. Each point represents an ASV plotted by its frequency and abundance across hosts within a location. All sampling locations indicated Neutral dispersal (Cabo Douglas R^2=0.842, Punta Espinoza R^2=0.832, Rabida R^2= 0.765).

**
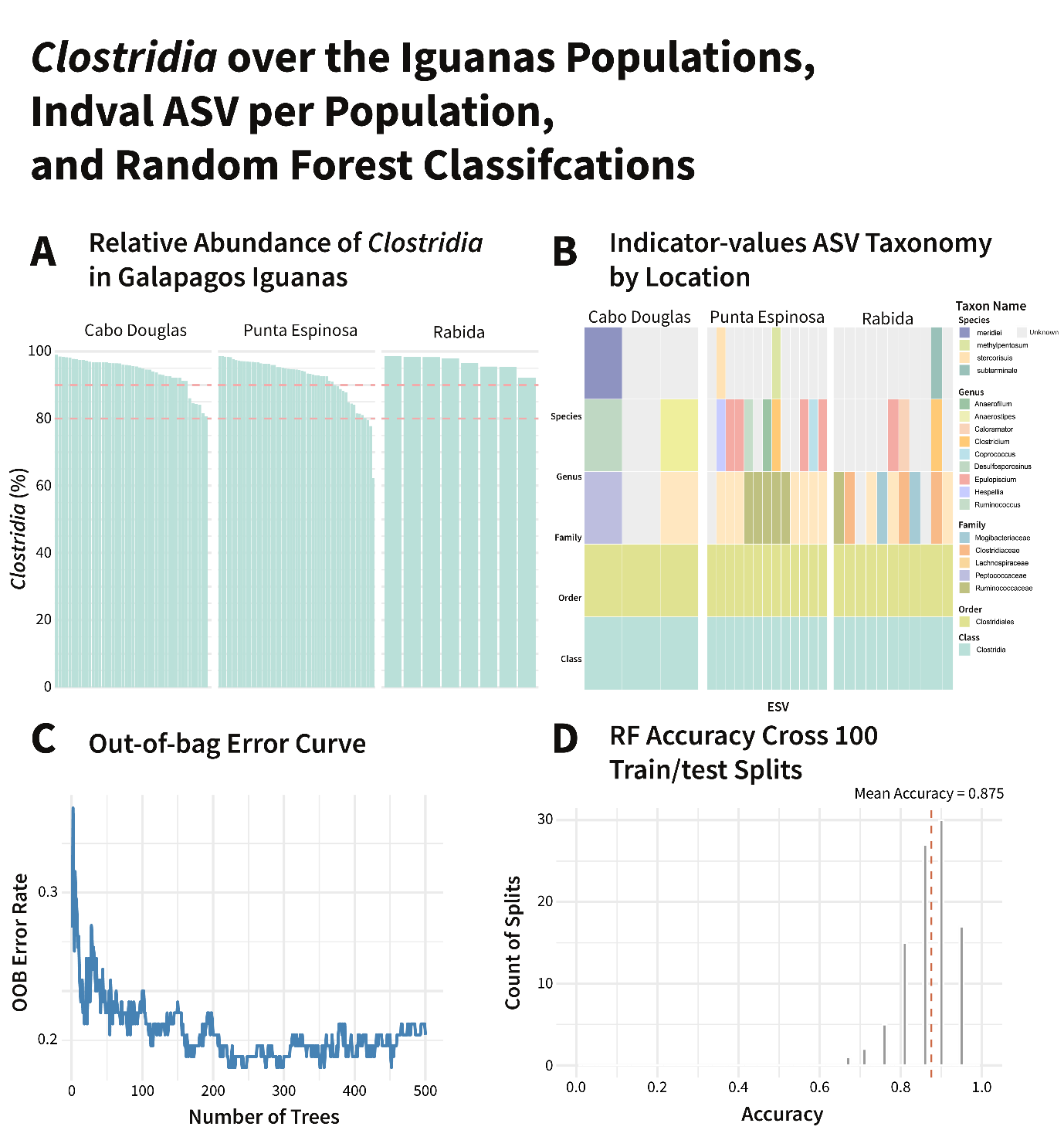
**

**Figure S3. Clostridia dominate the marine iguana fecal microbiota, with indicator taxa and machine learning models revealing predictive geographic signatures.**

A. Relative abundance of the bacterial class *Clostridia* across all samples. This panel illustrates that *Clostridia* are the predominant taxa in the marine iguana fecal microbiota, comprising the majority of the microbial community in most individuals.

**B.** Full taxonomic annotation of indicator ASVs identified through IndVal (Indicator Value Analysis). The y-axis displays taxonomic classifications from class to species level for ASVs significantly associated with specific locations, highlighting taxonomic resolution and ecological relevance.

**C.** Out-of-bag (OOB) error rate curve from the random forest model predicting the geographic origin of marine iguana samples based on microbiota composition. The error rate decreases and stabilizes with the inclusion of more trees, supporting the robustness of the classifier.

**D.** Distribution of prediction accuracy from 100 repeated random train/test splits of the random forest model. The accuracies are approximately normally distributed with a mean prediction accuracy of 87.5%, indicating strong and reproducible predictive power of the fecal microbiota composition for identifying marine iguana location.

**
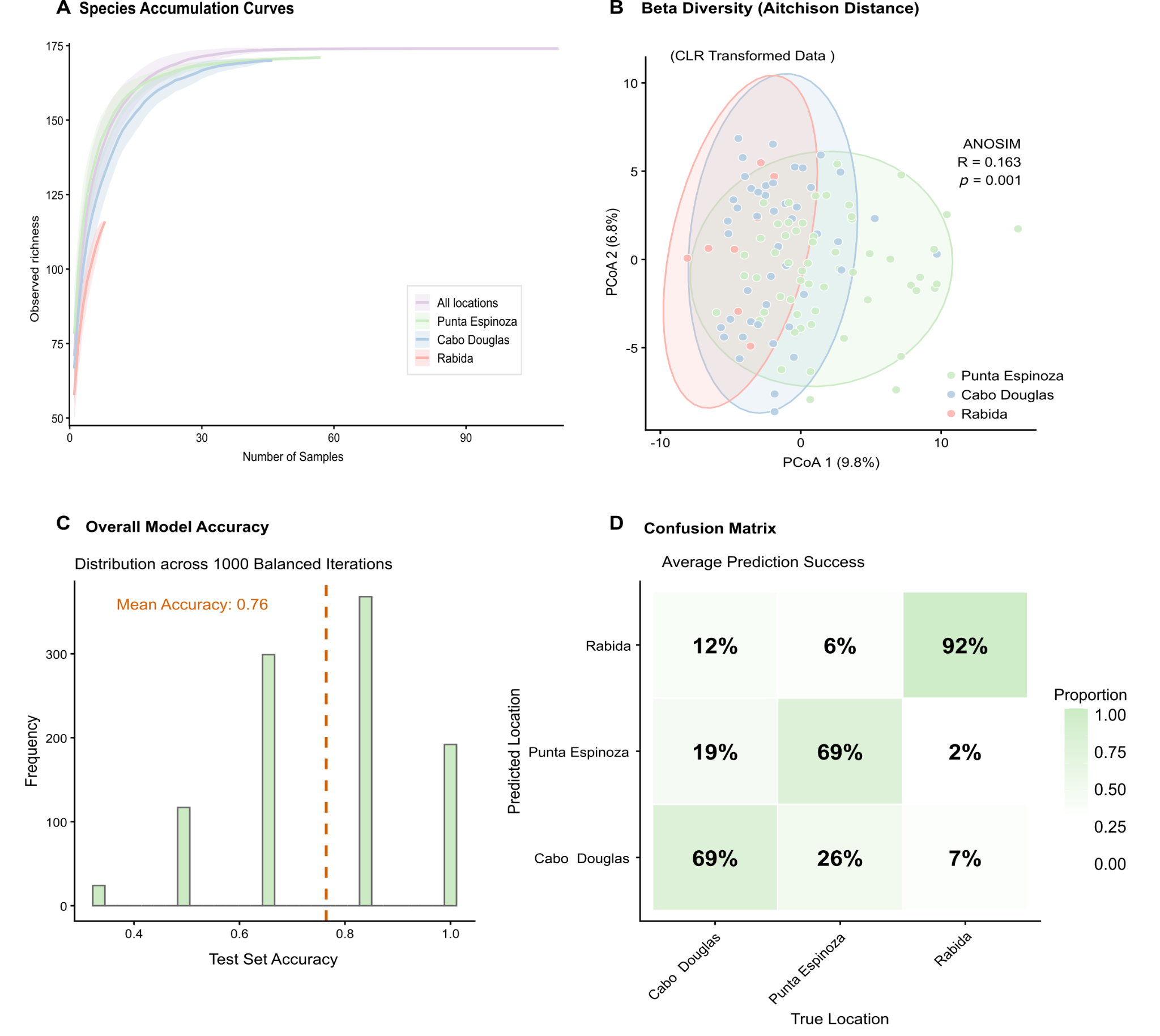
**

**Figure S4. Robustness analysis of sequencing depth, compositional beta diversity, and classifier performance under balanced sampling.**

**A.** Species accumulation curves for each sampling location and the aggregate dataset. The curves demonstrate that while the aggregate dataset ("All Locations") and the larger populations (Punta Espinoza, Cabo Douglas) reach saturation, the smaller Rabida cohort (n=8) remains unsaturated, reflecting sample size limitations.

**B.** Principal Coordinate Analysis (PCoA) based on Aitchison distance (Center Log-Ratio CLR transformed abundances). This compositionally aware metric confirms the beta-diversity patterns observed with UniFrac, showing weak but statistically significant geographic clustering (ANOSIM R=0.163, p=0.001). Ellipses represent 95% confidence intervals.

**C.** Distribution of Random Forest model accuracy across 1,000 iterations. To address class imbalance, the larger groups (Punta Espinoza, Cabo Douglas) were iteratively downsampled to match the Rabida sample size (n=8). The dashed line indicates the mean classification accuracy (0.76) across all balanced iterations.

**D.** Confusion matrix showing the average prediction success of the balanced Random Forest models. Values represent the proportion of true samples (x-axis) correctly assigned to their predicted class (y-axis). Notably, balancing the data resolves the bias against the minority class observed in the full model, with Rabida samples now being correctly classified in 92% of iterations.
